# New Appliances and Things Medical

**Published:** 1902-06-28

**Authors:** 


### NEW APPLIANCES AND THINGS MEDICAL.

**[We shall be glad to receive at our Office, 28 & 29 Southampton Street, Strand, London, W.C., from the manufacturers, specimens of all new preparations and appliances which may be brought out from time to time.]**

### SANITARY FEEDING BOTTLE.

(Hockin, Wilson & Co., 13 to 16 New Inn Yard, 186a Tottenham Court Road, London, W.; also at Manchester.)

The sanitary feeding bottle which is manufactured by Messrs. Hockin, Wilson & Co., and is known as the "Ever-Sweet" is a combination of the most highly appreciated improvements which have been effected in this class of bottle. It is a boat-shaped bottle with a flat bottom for standing. It is open at both ends so that it can be flushed through by applying it to the tap. At one end is fixed a most approved variety of rubber nipple, and at the other end is an india-rubber cap which contains a pin hole, and which can therefore act as an efficient valve for the admission of air. The bottle is further graduated in ounces, which are unusually accurate. We commend the "Ever-Sweet" feeding bottle as a practical and sanitary variety for general nursing use.

### McDOUGALL'S CARBOLIC AND SULPHUROUS DISINFECTANTS.

(McDougall Brothers, 68 Port Street, Manchester; 10 Mark Lane, London, E.C.)

More than fifty years ago Mr. Alexander McDougall, in conjunction with Dr. Angus Smith, directed their energies to the utilisation of bodies belonging to the carbolic or phenol series, as practical disinfectants for domestic or public use. It is to their investigations that we owe many of the greatest improvements in the methods of sanitary disinfection, and the antiseptics manufactured by the firm of MacDougall Brothers have enjoyed a world-wide reputation and an ever-increasing demand ever since their scientific and laboratory experiments took the practical shape of commercial realisation. It is impossible to specify the various powders, soaps and fluids which represent the many varieties of antiseptics manufactured by this firm. We would, however, particularly call attention to the soluble disinfecting powder, which contains sulphurous as well as carbolic acid in combination. The powder is entirely soluble and when so dissolved in water liberates the sulphurous acid from combination and thus exercises a doubly powerful antiseptic and disinfectant action. It is a valuable powder for water-closets, sewers, and drains of all kinds. McDougall's disinfectant washing oil is another preparation possessing novel features. This oil or liquid soap is strongly to be recommended for the washing of linen, etc., from contaminated sources, or at fever hospitals. It is supplied in casks or drums in bulk, and consists of four qualities: *i.e.*, carbolic, eucalyptus, sanitary pine, and almond. Non-poisonous purifiers are also a speciality of this firm, and take the form of pine-eucalyptus, and carbolate liquid disinfectants; these preparations are safe and efficient for household use, and may, especially in the case of the fluid carbolate, be used for gargles or internal medication. The various toilet and domestic soaps, tooth powders, urinal blocks, etc., included in the firm's list of specialities are well worthy the attention of those who realise the importance of cleanliness and sanitary methods.

